# Factors Associated with Anemia and Iron Deficiency during Pregnancy: A Prospective Observational Study in Japan

**DOI:** 10.3390/nu16030418

**Published:** 2024-01-31

**Authors:** Sarasa Habe, Megumi Haruna, Kaori Yonezawa, Yuriko Usui, Satoshi Sasaki, Takeshi Nagamatsu, Megumi Fujita, Yoshiko Suetsugu, Riko Ohori, Moeko Tanaka, Satoko Aoyama

**Affiliations:** 1 Department of Midwifery and Women’s Health, Division of Health Sciences and Nursing, Graduate School of Medicine, The University of Tokyo, Tokyo 113-0033, Japan; sarasahabe-9@g.ecc.u-tokyo.ac.jp (S.H.); kaoriyone@m.u-tokyo.ac.jp (K.Y.); yusui@g.ecc.u-tokyo.ac.jp (Y.U.); rohori@g.ecc.u-tokyo.ac.jp (R.O.); tanaka-moeko48526@g.ecc.u-tokyo.ac.jp (M.T.); aoyama-satoko125@g.ecc.u-tokyo.ac.jp (S.A.); 2 Global Nursing Research Center, Graduate School of Medicine, The University of Tokyo, Tokyo 113-0033, Japan; 3 Department of Social and Preventive Epidemiology, School of Public Health, The University of Tokyo, Tokyo 113-0033, Japan; stssasak@m.u-tokyo.ac.jp; 4 Department of Obstetrics and Gynecology, Faculty of Medicine, The University of Health and Welfare, Chiba 286-8520, Japan; tnag-tky@umin.ac.jp; 5 Department of Clinical Nursing, Graduate School of Medical Science, Yamagata University, Yamagata 990-9585, Japan; f.megumi@med.id.yamagata-u.ac.jp; 6 Department of Health Sciences, Graduate School of Medical Sciences, Kyushu University, Fukuoka 812-8582, Japan; suetsugu.yoshiko.742@m.kyushu-u.ac.jp

**Keywords:** ferritin, gestational anemia, hemoglobin, iron deficiency, nutrient intake

## Abstract

Gestational anemia (GA) is a global health concern with a remarkably high prevalence in Japan, which is associated with various maternal and neonatal outcomes. This study aimed to explore whether GA and non-anemic iron deficiency (NAID) during the third trimester is associated with maternal characteristics, nutrient intake, low birth weight (LBW), and preterm birth. Participants were categorized into GA, NAID, and normal groups, based on serum ferritin and hemoglobin levels. Nutrient intake was assessed using the Brief Diet History Questionnaire. Data from 317 pregnant women were analyzed, including 110 (34.7%), 151 (47.6%), and 56 (17.6%) women in the GA, NAID, and normal groups, respectively. Factors associated with GA included being multipara (*p* < 0.001) and not taking any type of iron supplements in the third trimester (*p* = 0.043). The normal group had a significantly higher proportion of preterm birth and LBW than the GA and NAID groups. The GA group had a significantly higher energy intake than the normal group (*p* = 0.044). Overall, energy and micronutrient intake were significantly below the estimated average requirement in the dietary reference intakes for Japanese. Health care professionals need to consider nutritional advice that can prevent GA by focusing on overall micronutrients, not just energy intake.

## 1. Introduction

Gestational anemia (GA) is a global women’s health concern. Based on a survey conducted by the World Health Organization (WHO) in 2019, 23.4% of pregnant women aged 15–49 years in Japan and 11.5–19.2% in Europe and North America were reported to have anemia [1]. GA can lead to poor neonatal and maternal outcomes, such as low birth weight (LBW), preterm birth, and maternal depression during pregnancy and postpartum [2,3]. Additionally, the main symptom of GA is fatigue; therefore, GA can lead to low physical activity [4]. Pregnant women are more prone to develop GA in the third trimester because the demand for fetal iron is highest [5]. Thus, pregnant women tend to have iron deficiency, which is the most common cause of anemia. When the body’s iron requirements are not met, its iron stores are reduced and non-anemic iron deficiency (NAID) [6] occurs. Eventually, once iron stores are mostly depleted, iron-deficiency anemia develops. While it is important to focus on GA, it is also necessary to pay attention to NAID to prevent iron-deficiency anemia.

In Japan, pregnant women need to intake iron from food, and symptomatic treatment for GA is common. After 9 weeks of gestation, iron supplements should be prescribed if the hemoglobin (Hb) level is <11 g/dL and healthcare professionals advise having a generally iron-rich diet [7]. In other developed countries, preventive treatment for GA, especially intermittent iron supplementation, has become popular [8]. Intermittent iron supplementation reduces GA. However, it is accompanied by digestive symptoms as an adverse effect, and information about improvements in delivery outcomes is lacking [8]. There is a lack of evidence that recommendations of other countries are applicable to pregnant women in Japan. This is because the adverse effects of GA are sensitive to the perinatal care environment, economic situation, and nutritional status of the country [9]. GA may be preventable via nutritional advice before prescribing iron supplements.

Although the situation may be different for other countries, in Japan, the risk factors for GA have not been thoroughly investigated. Conversely, in other countries, several risk factors for GA have been identified, including increased maternal age, multiparity, iron deficiency, low family income [10], history of alcohol intake, and irregular menstrual cycles in pre-pregnancy [11]. One significant risk factor for iron-deficiency anemia is a lack of iron intake from food [12]. Additionally, deficiencies in erythropoiesis-related micronutrients (folic acid and vitamin B12), inadequate nutrient intakes of protein, carbohydrates, fats, minerals, and other vitamins, and inadequate energy intake may lead to the development of GA [7,13]. GA is globally associated with poverty and often results from insufficient iron intake because of inadequate dietary intake. Japan is unique in the occurrence of insufficient iron intake despite adequate food availability. According to the National Health and Nutrition Survey (NHNS), the daily iron intake per person in Japan decreased from 13.4 mg in 1975 to 7.6 mg in 2020 [14,15]. This has been occasioned by a drastic change in the eating style of the Japanese people from a Japanese diet to a Western diet over the past 50 years. The Japanese diet comprises mainly rice, which is the staple food, as well as seafoods, meat, and vegetables. The Japanese diet has a balanced nutritional content and is considered an ideal diet similar to the Mediterranean diet [16]. In contrast, the Western diet has an imbalanced nutritional composition, mainly containing excessive fat and proteins [17]. As a result, the iron intake of Japanese people has decreased and the number of Japanese people with anemia has increased over time. According to 2018 NHNS [14] and 2019 WHO [18] surveys, 19.0% of non-pregnant women aged 15–49 years in Japan have anemia.

Therefore, this study aimed to explore whether GA and NAID during the third trimester are associated with maternal characteristics, nutrient intake, LBW, and preterm birth. By identifying these factors, healthcare professionals can provide more effective nutritional advice.

## 2. Materials and Methods

### 2.1. Study Design and Study Population

This prospective observational study was part of the Japan Pregnancy Eating and Activity Cohort (J-PEACH) Study [19]. From March 2020 to August 2021, pregnant women attending an obstetrics outpatient clinic at a tertiary emergency medical facility in Tokyo, Japan were recruited. However, the recruitment was temporarily halted from April 2020 to July 2020 and from November 2020 to March 2021 owing to the impact of the coronavirus disease 2019 (COVID-19) pandemic. Women whose blood samples were collected during the third trimester and who completely responded to the third trimester questionnaire were included. Women with multiple births, who reported unrealistic energy intake and answered the questionnaire too late (after delivery) were excluded from the analysis. The questionnaire included information on maternal characteristics, nutrient intake, and physical activity from 35 weeks of pregnancy. Paper-based questionnaires were initially used; however, web-based questionnaires were administered after the COVID-19 pandemic began.

### 2.2. Definition of Categories

#### 2.2.1. Categories of Serum Iron Status

Only Hb and serum ferritin were used to categorize participants. We did not categorize thalassemia and pernicious anemia. This is because, when GA is diagnosed, it is initially treated as iron-deficiency anemia in Japan. If iron supplementation proves ineffective, investigations into causes other than iron-deficiency anemia are then undertaken, and only at that point might thalassemia and pernicious anemia be diagnosed. Serum ferritin levels have been recognized as an effective screening tool for iron deficiency and the most commonly used threshold of serum ferritin for the diagnosis of iron deficiency is <12 ng/mL [5]. The serum iron status of pregnant women was classified into three groups: the GA group (Hb levels < 11 g/dL) [8], the NAID group (Hb levels ≥ 11 g/dL and serum ferritin levels < 12 ng/mL), and the normal group (Hb levels ≥ 11 g/dL and serum ferritin levels ≥ 12 ng/mL).

#### 2.2.2. Prescription of Iron Supplements and Dietary Iron Supplements Use

Iron supplements prescribed by doctors during the first, second, and third trimesters were collected from the patients’ medical records. Only the prescribed supplements used before blood sample collection were categorized as prescribed iron supplements. Information on dietary iron supplement (not prescribed iron supplements including multi-micronutrient supplements) taken in the preceding month was obtained via the third trimester questionnaire. Responses were classified as “prescribed iron supplements”, “taking only dietary iron supplements”, or “not taking either”.

### 2.3. Data Collection

#### 2.3.1. Participants’ Characteristics and Neonatal Outcomes

Parity, maternal age at birth, maternal pre-delivery weight, neonatal birthweight, birth height, head circumference, chest circumference at birth, and infant sex were extracted from the patients’ medical records after delivery. Definitions of terms are as follows: preterm birth, delivery before 37 weeks; LBW, birth weight < 2500 g; small for gestational age, birth weight below 10th percentile for gestational age. Pre-delivery weight was defined as the last weight measurement before delivery. Maternal pre-pregnancy weight, Edinburgh Postpartum Depression Scale (EPDS) score in the third trimester (T3), physical activity, history of drinking and smoking status, marital status, educational level, working status in T3, and family income were collected through questionnaires. Gestational weight gain (GWG) was calculated by subtracting the self-reported pre-pregnancy weight from the pre-delivery weight. The cutoff value of EPDS in the third trimester was 9 points. Physical activity during the third trimester was assessed using the Japanese version of the Pregnancy Physical Activity Questionnaire (PPAQ-J) 2020 [20]. PPAQ-J 2020 is a Japanese translation of the original PPAQ [21] and modified to adopt the current lifestyle. Physical activity is calculated using the metabolic equivalents (METs). The updated PPAQ is used as a reference [22]. Reliability and validity of the PPAQ, updated PPAQ, and PPAQ-J have already been verified [21,22,23].

#### 2.3.2. Blood Sampling and Biomarkers

Fasting blood samples were collected from participants at 34–39 weeks of gestation. Blood samples were collected in serum separation tubes (VENOJECT2; Terumo, Tokyo, Japan). After collection, the serum was centrifuged at 3500 rpm for 5 min at −80 °C, and then stored in a −80 °C freezer. The samples were evaluated within 17 months. Blood samples were used to measure ferritin levels. The biomarker measurements were performed in a contracted laboratory (SRL Inc., Tokyo, Japan). Ferritin levels were measured using the chemiluminescent enzyme immunoassay method.

Biomarker data, which included Hb, Hct, MCV, mean corpuscular hemoglobin (MCH), mean corpuscular hemoglobin concentration (MCHC) and red cell distribution width (RDW), were obtained from the patients’ medical records. Hb levels in the first trimester (T1) were measured for up to 13 weeks, and Hb levels in the second trimester (T2) were measured between 18 weeks and 27 weeks. Hb, Hct, MCV, MCH, MCHC, and RDW in T3 were collected close to 35 weeks of gestation.

#### 2.3.3. Nutrient Intake

The Brief Self-Administered Diet History Questionnaire (BDHQ) is a validated questionnaire for Japanese pregnant women that has been used to measure macronutrients and micronutrients [24]. Data from the questionnaire regarding the foods based on the Japanese Standard Tables of Food Composition [25] consumed by participants over the preceding month were collected and analyzed. According to previous studies [26], nutrients related to erythropoiesis were included in the analysis. For the analysis, the intakes of calcium; iron; zinc; vitamins D, B1 B2, B6, B12, and C; folate; and dietary fiber intake were energy adjusted using the density method (/1000 kcal) to reduce individual measurement errors. We excluded participants who reported extremely unrealistic energy intake; that is, the reported energy intake was less than one-half of the energy requirement for the lowest physical activity level or more than 1.5 times the energy requirement for moderate physical activity, based on the dietary reference intakes for Japanese 2020 guidelines [27].

### 2.4. Statistical Analysis

Continuous variables with a normal distribution are presented as the mean ± the standard deviation and were subjected to one-way analysis of variance, followed by post hoc comparisons using Tukey’s test. Physical activity and nutrient intakes do not follow a normal distribution, and are presented as the median (interquartile range) and subjected to the Kruskal–Wallis test, followed by post hoc comparison with Bonferroni correction. Categorical variables are presented as the frequency and percentage and were analyzed using the chi-squared test. For maternal characteristics, neonatal outcomes, and nutrient intake, pregnant women were categorized, based on their serum iron status, into the GA, NAID, or normal group. Additionally, physical activity and nutrient intake were analyzed separately from primipara and multipara. Thus is because parity is related to physical activity [28] and dietary patterns [29]. Statistical significance was set at a two-sided *p*-value < 0.05. All analyses were conducted using SPSS version 29.0 for Microsoft Windows (IBM Corp., Armonk, NY, USA). Sample size was calculated using G*Power version 3.1.9.7 [30]. Maternal characteristics were indicated as an outcome. The sample size was calculated at the 5% level with a power allocation ratio of 0.25. The estimated sample size was 252.

## 3. Results

### 3.1. Participants

**Figure 1:**
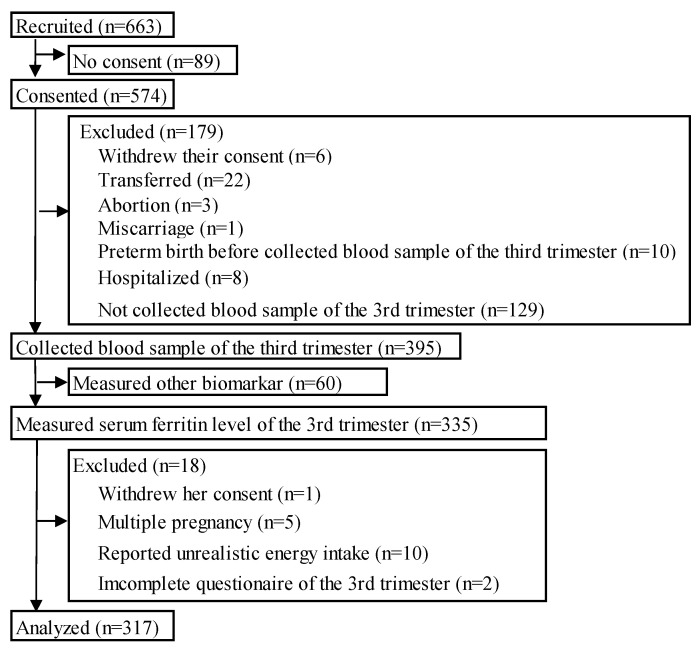
Flowchart of present study.

In total, 574 women consented to participate in the study. Blood samples were collected from 395 women in T3. Serum ferritin levels were measured in samples from 335 women. Eighteen women were excluded, resulting in the final analysis of data obtained from 317 women (Figure 1).

### 3.2. Biomarkers of the Participants

Table 1 shows the characteristics of the 317 women included in the analysis. The distribution of participants among the GA, NAID, and normal groups was 110 (34.7%), 151 (47.6%), and 56 (17.7%), respectively.

### 3.3. Characteristics of the Participants

Overall, 193 (60.9%) women were primiparous and 124 (39.1%) were multiparous. The proportion of multiparous women was significantly higher in the GA group than in the other two groups. The mean age of women at birth was 35.0 ± 4.1 years, with no significant differences among the groups and parity. The pre-pregnancy BMI and GWG were not significantly different. Smoking was more prevalent in the NAID group (15.9%) than in the GA (6.4%) and normal groups (5.4%) (*p* = 0.018). The overall physical activity of all participants was 121.8 METs (IQR: 92.5–164.5), with no significant difference among the three groups (*p* = 0.755). The physical activity for 191 primiparous women was 112.5 METs (IQR: 83.1–150.9), and that for 123 multiparous women was 141.1 METs (IQR: 107.8–187.1). Compared with primiparous women, multiparous women showed significantly higher physical activity (*p* < 0.001). No significant differences among the groups were observed in EPDS score, history of drinking, marital status, educational level, working status, and family income.

### 3.4. Prescribed Iron Supplements and Dietary Supplements Use

At T1, only one participant in the GA group was using prescribed iron supplements. At T2, the proportion of participants using prescribed iron supplements was significantly higher in the GA group than in the NAID group. At T3, among the 317 women, 41 (12.9%) women used prescribed iron supplements, 156 (49.2%) women used only dietary iron supplements, and 120 (37.9%) women did not take either. In the GA group, the number of women who did not take any supplements was significantly higher. Multiparous women who took only dietary supplements were significantly lower between parity (*p* = 0.022).

### 3.5. Neonatal Outcomes

The number of preterm births among all participants was 11 (3.4%) and was significantly higher in the normal group. The frequency of LBW was 36 (11.3%) among all participants and was significantly higher in the normal group than in the other two groups. When excluding preterm births, the birth weight was similar among the groups: 3109 ± 344 g in the GA group, 2989 ± 367 g in the NAID group, and 3039 ± 370 g in the normal group (*p* = 0.037). The number of small for gestational age was 14 (4.4%) among all participants and was significantly higher in the normal group.

### 3.6. Nutrient Intake among the GA, NAID, and Normal Groups

Table 2 shows the comparison of nutrient intake among women who were classified via their serum iron status. The overall energy intake of all participants was 1518 kcal (IQR: 1298–1749). Energy intake for the GA, NAID, and normal groups was 1581 kcal (IQR: 1353–1880), 1524 kcal (IQR: 1280–1730), and 1404 kcal (IQR: 1287–1640), respectively. The results of multiple comparisons, based on Bonferroni corrections, revealed that the normal group had a significantly lower energy intake than the GA group (*p* = 0.044). No significant differences existed among the three groups in the intakes of macronutrients and micronutrients.

### 3.7. Nutrient Intake of Primipara and Multipara among the GA, NAID, and Normal Groups

Table 3 shows nutrient intake, based on iron status, of primipara, and Table 4 shows that of multipara. The energy intake for the 193 primipara was 1478 kcal (IQR: 1286–1721) and iron intake was 4.1 mg/1000 kcal (IQR: 3.6–4.7). The energy intake for the 124 multipara was 1557 kcal (IQR: 1314–1893) and the iron intake was 3.9 mg/1000 kcal (IQR: 3.5–4.4). Compared with primipara, multipara had a significantly higher energy intake, but no significant difference was observed in iron intake (energy: *p* = 0.045; iron: *p* = 0.353). Among multipara, compared with the normal group, the GA group had a significantly lower intake of fat (*p* = 0.044) and the NAID group had a significantly lower intake of vitamin B12 (*p* = 0.048). Carbohydrate levels were significantly higher in the GA group than in the NAID and normal groups (*p* = 0.031 and *p* = 0.028, respectively). No significant differences existed among the three groups in the intake of other nutrients.

## 4. Discussion

### 4.1. Key Results

The novelty of this study is in the fact that it focuses on the accurate assessment of serum iron status in the third trimester of pregnancy based on serum ferritin levels. This study focuses not only on GA but also high-risk GA. More than 80% of participants had GA or were at high risk of developing GA. Multiparity was associated with developing GA in the third trimester. The GA and NAID groups had a significantly lower proportion of preterm births and LBW than normal group. Having GA and NAID did not indicate induced preterm birth and LBW. By nutrient intake, energy intake was highest in the GA group, followed by NAID and normal groups.

### 4.2. Maternal Characteristics and Neonatal Outcomes

This study found a higher prevalence of GA in pregnant women (34.7%) compared to that found in the WHO survey conducted in Japan in 2019 [1]. There are few previous studies focusing on ferritin [31]; this showed that 261 (82.3%) participants had GA or were high-risk pregnant women, which was surprisingly high for an industrialized country. The annual incidence of pernicious anemia is 1/100,000 and the gene frequency for β- and α- thalassemia has been shown to be 1/1000 and 1/3500, respectively, in the Japanese population [7]. Although the gene frequency for thalassemia is relatively high, based on medical records and other blood data, no pregnant women in this study were found to have pernicious anemia or thalassemia. Thus, it is suggested that participants with GA in this study likely have iron-deficiency anemia. Approximately 90% of the participants had a family income of at least 5 million yen. Thus, they belonged to the high-family-income group, as the median household income in Japan is 4.23 million yen [32]. The educational level and family income of most participants in this study were higher, and are known as two protective factors of GA because they promote dietary diversity [33]. However, many women in this study were 35 years or older and had low pre-pregnancy BMI, factors demonstrated by previous studies as potentially influencing the development of GA [10,33,34]. In the present study, the average pre-pregnancy BMI was 21.0 kg/m^2^, which reflects the characteristics of Japanese women of reproductive age. According to the NHNS, non-pregnant women aged 20–29 years and 30–39 years in Japan had an average pre-pregnancy BMI of 21.0 kg/m^2^ and 21.7 kg/m^2^, respectively [14]. The reasons for the high prevalence of low pre-pregnancy BMI among young Japanese women are complex, but the most significant reason is that many young Japanese women believe that being thin is beautiful.

Multiparous women had the highest rates of GA, a finding consistent with studies conducted in other countries [31,35]. It is not yet clearly understood why multiparous women are more prone to GA. A previous study suggested that multiparous women lack sufficient time to recover from the nutritional burden of their previous pregnancy [31,35]. The present study does not implicate that age acted as a confounder in the relationship between parity and GA, because there was no significant difference in maternal age between primipara and multipara.

The present study revealed a significantly higher incidence of preterm birth and LBW in the normal group. Previous studies [36,37] have shown that maternal Hb level has a U-shaped relationship with the incidence of preterm birth and LBW. Severe anemia (Hb level ≤ 8.5 g/dL) results in a higher incidence of preterm birth and LBW [38]. The mean Hb level of the GA group in this study was 10.2 ± 0.5 g/dL. Studies have shown that women with a Hb level in this range have a relatively low incidence of preterm birth and LBW, and our study shows the same results [2,35,39]. No participants in this study had an Hb level ≤ 8.5 g/dL. Therefore, the left extreme of the U-shaped curve cannot be observed. Existing evidence for the trimester of anemia that influences the incidence of preterm birth and LBW is inconsistent. In this study, we focused on LBW and preterm birth as neonatal outcomes. A previous study [2] suggested that GA is also related to preeclampsia, delivery hemorrhage, and duration of labor. In future research, not only should Hb be monitored from the first trimester to postpartum period, but also serum ferritin. The association between GA/NAID and other delivery outcomes need to be investigated.

### 4.3. Prescribed/Dietary Iron Supplements Use and Biomarkers

In the GA group, a significant proportion of women did not take prescribed or dietary iron supplements. Considering the possibility of a future clinical diagnosis in the GA group, they would be prescribed iron supplements. The present study suggests that symptomatic treatment might be adequate because the GA group had lower rates of preterm birth and LBW. Intermittent iron supplementation, which is known as a popular means of preventing GA, may result in a Hb level greater than 13.0 g/dL [8]. It is noteworthy that Hb levels < 13.0 g/dL can increase the risks of LBW [37,38].

Multi-micronutrient supplements are commonly used in developed countries [40,41]. However, previous studies have not provided consistent evidence on the effectiveness of dietary iron supplements, including multi-micronutrient supplements [42,43]. A cohort study conducted in Norway [42] showed that women taking multi-micronutrient supplements had higher serum ferritin than women with no multi-micronutrient supplementation. In addition, a Cochrane Review [43] showed that multi-micronutrient supplements reduce the incidence of preterm births and LBW, but have no positive effect on GA [44]. Additionally, multi-micronutrient supplements may contain minerals that hinder iron absorption [44]. Therefore, pregnant women should not rely solely on dietary iron supplements for the prevention of GA. The present study could not consider medication adherence. Future research is needed to explore the effects of medication interruptions or missed doses.

### 4.4. Nutrient Intake and Biomarkers

The energy intake for all groups in this study was below the estimated energy requirements for pregnant women aged 30–49 in the third trimester [27]. The average energy intake of non-pregnant women aged 30–49 years was approximately 1600 kcal [14], which falls below the estimated energy requirements. Many participants had low pre-pregnancy BMI and may have had even lower energy intake at pre-pregnancy. Furthermore, they may not have increased their energy intake sufficiently after pregnancy. The nutrient intake of participants was insufficient. The GA group had a significantly higher energy intake than the normal group; this high energy intake was associated with the larger number of multiparous women in the GA group. The literature suggests that multiparous women have a higher energy intake than primiparous women [45]. Energy intake was positively associated with total physical activity levels [46]. The present study showed that multiparous women had a significantly higher energy intake associated with higher total physical activity. These findings are consistent with those from previous studies [28]. The difference in nutrient intake between primiparous women and multiparous women may be related to dietary behavior. In the future, it will be necessary to focus not only on nutrient intake, but also on eating behavior and eating habits. Iron intake was notably lower than that in the dietary reference intakes for Japanese guidelines, in which the estimated average requirement for pregnant women aged 30–49 in the third trimester is 14.5 mg [27]. Similar trends in iron intake below the estimated average requirement have been observed in some Western countries [47]. The present study suggests that a higher energy intake did not necessarily result from an iron-rich diet and was unable to prevent GA. Moreover, it is important to note that the intake of micronutrients other than iron was below the estimated average requirement for our participants [27]. Considering that their pre-pregnancy BMI was low, there is a possibility that these women had chronic nutrient deficiency. The association of chronic nutrient deficiency with GA has been identified [48].

Healthcare professionals need to consider nutritional advice that can prevent GA by focusing on overall micronutrients and not merely energy intake. Specifically, healthcare professionals should promote the consumption of animal foods, such as red meat, which is rich in heme iron [12], and fruits and green vegetables, which are rich in vitamin C [13,42,49]. Pregnant women should also avoid coffee and tea, as caffeine inhibits iron absorption [42].

### 4.5. Limitations and Strengths

This study had three main limitations. First, data on pre-pregnancy anemia status, menstrual cycle, menstrual volume, and birth interval were not collected, potentially affecting the assessment of factors related to GA [35,50]. Second, the use of dietary iron supplements was self-reported, which introduced the possibility of under-estimation among pregnant women who may have unknowingly used iron-containing products. Third, data on whether participants had previously received nutritional guidance were not collected, which could have influenced their nutrient intake. However, this study has three strengths. First, serum ferritin levels were measured to define NAID and characteristics of NAID were described. Although serum ferritin is an effective screening tool for iron deficiency [5], only a few studies have measured the serum ferritin of participants. Second, the study design was a prospective observational study, which minimized selection bias as much as possible. Third, the use of the BDHQ was a result of the careful monitoring of nutrient intakes over the preceding month.

## 5. Conclusions

Among the study participants, more than 80% of women had GA or were at high risk of GA. Factors associated with GA included being multiparous and not taking any iron supplement in the third trimester. However, GA did not affect preterm birth and LBW. The GA group had significantly higher energy intake than the normal group. Energy and micronutrient intakes were significantly below the estimated average requirement in the dietary reference intakes for Japanese. The results of this study suggest the need for healthcare professionals to advise pregnant women to increase overall micronutrient intake.

## Figures and Tables

**Table 1:** Characteristics of participants and neonatal outcomes.

		Gestational Anemia	Non-Anemic Iron Deficiency	Normal	
		n = 110 (34.7%)	n = 151 (47.6%)	n = 56 (17.7%)	
		Mean ± SD or n (%)	Mean ± SD or n (%)	Mean ± SD or n (%)	*p*
Maternal biomarker					
T1	Hb (g/dL) (n = 156)	13.3 ± 4.9	13.1 ± 0.8	13.7 ± 4.3	0.776
T2	Hb (g/dL) (n = 227)	11.3 ± 0.8	11.9 ± 0.8	11.7 ± 1.0	<0.001
T3	Hb (g/dL)	10.3 ± 0.5	11.8 ± 0.7	12.3 ± 0.9	<0.001
T3	Ferritin (ng/L)	8.2 ± 8.0	7.1 ± 2.0	20.6 ± 13.9	<0.001
T3	Hct (%)	32.1 ± 1.4	35.8 ± 1.9	36.4 ± 4.1	<0.001
T3	MCV (fL)	88.7 ± 5.7	91.5 ± 4.5	94.2 ± 4.6	<0.001
T3	MCH (pg)	28.6 ± 2.4	30.2 ± 1.8	31.3 ± 1.9	<0.001
T3	MCHC (%) (n = 316)	32.2 ± 0.9	33.0 ± 0.8	33.2 ± 0.8	<0.001
T3	RDW (fL) (n = 297)	13.7 ± 1.7	13.0 ± 1.1	13.4 ± 1.2	<0.001
Maternal characteristics					
Parity	Primipara	55 (28.5)	93 (48.2)	45 (23.3)	<0.001 ^†^
	Multipara	55 (44.4)	58 (46.8)	11 (8.8)	
Maternal age		35.1 ± 4.0	35.1 ± 4.2	34.9 ± 4.6	0.921
Pre-pregnancy BMI		21.2 ± 3.0	21.4 ± 3.8	20.6 ± 2.8	0.284
GWG		9.1 ± 3.7	8.6 ± 3.5	7.8 ± 2.7	0.060
EPDS score	≥9 points	16 (29.1)	29 (52.7)	10 (18.2)	0.614 ^†^
	<9 points	94 (35.9)	122 (46.6)	46 (17.5)	
History of drinking (n = 309)					
	No	32 (33.0)	47 (48.5)	18 (18.5)	0.899 ^†^
	Yes	75 (35.4)	101 (40.1)	36 (24.5)	
Smoking status	No	103 (36.4)	127 (44.9)	53 (18.7)	0.018 ^†^
	Yes	7 (20.6)	24 (70.6)	3 (8.8)	
Marital status (n = 309)	Married	104 (34.1)	148 (48.5)	53 (17.4)	0.137 ^†^
	Not married	3 (75.0)	0 (0.0)	1 (25.0)	
Education (n = 304)	<University	20 (29.4)	36 (52.9)	12 (17.7)	0.588 ^†^
	≥University	85 (36.0)	111 (47.0)	40 (17.0)	
Working status	Employed	10 (40.0)	12 (48.0)	3 (12.0)	0.700 ^†^
	Not-working/Unemployed	100 (34.2)	139 (47.6)	53 (18.2)	
Family income (n = 308)	<5 million	12 (38.7)	15 (48.4)	4 (12.9)	0.738 ^†^
(Japanese yen/year)	≥5 million	94 (33.9)	133 (48.0)	50 (18.1)	
Prescribed iron supplements	T1	1 (100)	0 (0.0)	0 (0.0)	0.389 ^†^
	T2	13 (59.1)	5 (22.7)	4 (18.2)	0.028 ^†^
Taking any type of iron supplements during T3					0.043 ^†^
	Prescribed iron supplements	12 (29.3)	18 (43.9)	11 (26.8)	
	Only dietary iron supplements	48 (30.8)	75 (48.1)	33 (21.1)	
	Not taking either	50 (41.7)	58 (48.3)	12 (10.0)	
Neonatal outcomes					
Preterm birth (<37 weeks)		4 (36.4)	2 (18.2)	5 (45.4)	0.029 ^†^
Low birthweight (<2500 g)		3 (8.3)	14 (38.9)	19 (52.8)	0.004 ^†^
Birthweight (g)		3090 ± 354	2984 ± 369	2962 ± 471	0.041
Small for gestational age		1 (7.1)	7 (50.0)	6 (42.9)	0.014
Birth height (cm)		49.3 ± 1.8	48.9 ± 2.0	48.9 ± 2.5	0.208
Head/Chest ratio		1.0 ± 0.1	1.1 ± 0.0	1.1 ± 0.0	0.062
Sex	Male	51 (31.1)	84 (51.2)	29 (17.7)	0.335 ^†^
	Female	59 (38.6)	67 (43.8)	27 (17.6)	

There are missing data for T1 Hb, T2 Hb, History of drinking, Marital status, Education and Family income. SD, standard deviation; T1, 1st trimester; T2, 2nd trimester; T3, 3rd trimester; BMI, body mass index; GWG, gestational weight gain; EPDS, Edinburgh postpartum depression scale. One-way analysis of variance, ^†^ chi-square test.

**Table 2:** Maternal energy, macronutrient, and micronutrient intake among GA, NAID, and normal group.

		All		Gestational Anemia		Non-Anemic Iron Deficiency		Normal		*p*
		n = 317		n = 110		n = 151		n = 56		
		Median	(IQR)	Median	(IQR)	Median	(IQR)	Median	(IQR)	
Energy	(kcal/day)	1518	(1298–1749)	1581	(1353–1880)	1524	(1280–1730)	1404	(1287–1640)	0.047
Iron	(mg/1000 kcal)	4.0	(3.6–4.6)	3.9	(3.6–4.7)	4.1	(3.6–4.6)	4.0	(3.4–4.6)	0.819
Folic acid	(µg/1000 kcal)	164	(135–205)	165	(130–207)	163	(135–204)	163	(135–204)	0.989
Vitamin K	(µg/1000 kcal)	173	(127–241)	172	(120–248)	179	(132–233)	167	(125–251)	0.934
Vitamin B12	(µg/1000 kcal)	3.3	(2.4–4.7)	3.4	(2.5–4.7)	3.2	(2.4–4.5)	3.7	(2.1–5.3)	0.917
Vitamin D	(µg/1000 kcal)	4.8	(3.1–6.9)	4.8	(3.3–6.9)	4.9	(3.2–6.7)	4.4	(2.8–7.0)	0.634
Vitamin B1	(mg/1000 kcal)	0.4	(0.4–0.5)	0.4	(0.4–0.5)	0.4	(0.4–0.5)	0.4	(0.4–0.5)	0.922
Vitamin B2	(mg/1000 kcal)	0.7	(0.6–0.8)	0.7	(0.6–0.8)	0.7	(0.6–0.8)	0.7	(0.6–0.8)	0.874
Vitamin B6	(mg/1000 kcal)	0.7	(0.6–0.7)	0.6	(0.6–0.7)	0.6	(0.6–0.7)	0.7	(0.5–0.8)	0.831
Zinc	(mg/1000 kcal)	4.5	(4.2–5.0)	0.4	(0.4–0.5)	0.5	(0.4–0.5)	0.4	(0.4–0.5)	0.632
Vitamin C	(mg/1000 kcal)	56.8	(43.6–76.0)	56.5	(43.6–75.3)	55.0	(43.6–76.0)	60.8	(42.3–76.4)	0.875
Calcium	(mg/1000 kcal)	317	(263–383)	320	(259–390)	316	(270–374)	315	(254–395)	0.898
Dietary fiber	(g/1000 kcal)	6.7	(5.9–8.0)	6.8	(6.0–8.1)	6.7	(6.0–8.1)	6.5	(5.8–8.1)	0.730
Protain	(% energy)	14.6	(13.0–16.3)	14.5	(12.9–16.1)	14.7	(13.0–16.3)	14.8	(13.0–16.5)	0.532
Fat	(% energy)	30.1	(26.6–33.6)	29.2	(26.1–34.1)	30.0	(26.8–33.0)	31.1	(28.0–34.7)	0.286
Carbohydrate	(% energy)	54.0	(49.5–58.4)	54.4	(49.9–58.6)	54.2	(49.6–54.2)	52.1	(48.2–58.0)	0.264

IQR, Interquartile range. Kruskal–Wallis test.

**Table 3:** Maternal energy, macronutrient, and micronutrient intake of primipara.

		All		Gestational Anemia		Non-Anemic Iron Deficiency		Normal		*p*
		n = 193		n = 55		n = 93		n = 45		
		Median	(IQR)	Median	(IQR)	Median	(IQR)	Median	(IQR)	
Energy	(kcal/day)	1478	(1286–1721)	1540	(1354–1780)	1465	(1279–1708)	1375	(1256–1639)	0.106
Iron	(mg/1000 kcal)	4.1	(3.6–4.7)	4.0	(3.7–4.9)	4.3	(3.6–4.7)	4.0	(3.4–4.6)	0.512
Folic acid	(µg/1000 kcal)	171	(138–212)	174	(142–221)	175	(137–209)	164	(135–213)	0.805
Vitamin K	(µg/1000 kcal)	179	(136–247)	178	(145–256)	187	(135–242)	172	(131–257)	0.894
Vitamin B12	(µg/1000 kcal)	3.2	(2.4–4.6)	3.0	(2.2–4.7)	3.3	(2.4–4.5)	3.2	(2.0–4.9)	0.563
Vitamin D	(µg/1000 kcal)	4.4	(3.0–6.3)	4.2	(2.9–6.7)	5.0	(3.3–6.5)	3.9	(2.7–5.9)	0.110
Vitamin B1	(mg/1000 kcal)	0.5	(0.4–0.5)	0.5	(0.4–0.5)	0.5	(0.4–0.5)	0.4	(0.4–0.5)	0.922
Vitamin B2	(mg/1000 kcal)	0.7	(0.6–0.8)	0.7	(0.6–0.9)	0.7	(0.6–0.8)	0.7	(0.6–0.8)	0.969
Vitamin B6	(mg/1000 kcal)	0.7	(0.6–0.7)	0.7	(0.6–0.7)	0.7	(0.6–0.7)	0.6	(0.5–0.8)	0.903
Zinc	(mg/1000 kcal)	4.5	(4.2–5.0)	4.5	(4.1–5.0)	4.6	(4.2–5.0)	4.1	(4.1–5.0)	0.770
Vitamin C	(mg/1000 kcal)	59.1	(44.6–78.7)	59.8	(45.7–81.6)	58.3	(44.4–79.6)	59.1	(42.5–77.0)	0.927
Calcium	(mg/1000 kcal)	321	(276–392)	331	(278–415)	314	(288–375)	324	(254–423)	0.626
Dietary fiber	(g/1000 kcal)	6.7	(6.0–8.1)	7.0	(5.9–8.1)	6.7	(6.1–8.2)	6.5	(5.8–8.0)	0.586
Protain	(% energy)	14.7	(13.1–16.5)	14.6	(13.0–16.0)	14.8	(13.3–16.7)	14.1	(12.8–16.4)	0.595
Fat	(% energy)	30.6	(27.6–33.7)	29.3	(27.7–34.8)	30.9	(27.7–33.3)	30.3	(26.6–34.6)	0.983
Carbohydrate	(% energy)	53.5	(49.3–58.0)	54.2	(49.8–58.0)	53.2	(49.2–57.1)	53.5	(47.6–59.5)	0.837

IQR, Interquartile range. Kruskal–Wallis test.

**Table 4:** Maternal energy, macronutrient, and micronutrient intake of multipara.

		All		Gestational Anemia		Non-Anemic Iron Deficiency		Normal		*p*
		n = 124		n = 55		n = 58		n = 11		
		Median	(IQR)	Median	(IQR)	Median	(IQR)	Median	(IQR)	
Energy	(kcal/day)	1557	(1314–1893)	1594	(1339–1933)	1551	(1294–1747)	1450	(1330–2101)	0.715
Iron	(mg/1000 kcal)	3.9	(3.5–4.4)	3.8	(3.5–4.7)	4.0	(3.5–4.3)	4.1	(3.8–4.7)	0.598
Folic acid	(µg/1000 kcal)	156	(128–193)	161	(122–196)	152	(135–188)	157	(137–197)	0.920
Vitamin K	(µg/1000 kcal)	158	(117–221)	156.2	(110–236)	161	(126–210)	154	(116–252)	0.768
Vitamin B12	(µg/1000 kcal)	3.5	(2.5–4.7)	3.8	(2.8–4.7)	3.2	(2.2–4.6)	5.4	(3.4–6.4)	0.043
Vitamin D	(µg/1000 kcal)	5.3	(3.4–7.4)	5.6	(3.7–7.3)	4.8	(3.1–7.0)	7.1	(5.1–10.6)	0.105
Vitamin B1	(mg/1000 kcal)	0.4	(0.4–0.5)	0.4	(0.4–0.5)	0.4	(0.4–0.5)	0.4	(0.4–0.5)	0.875
Vitamin B2	(mg/1000 kcal)	0.7	(0.6–0.8)	0.7	(0.6–0.8)	0.7	(0.6–0.8)	0.7	(0.6–0.8)	0.979
Vitamin B6	(mg/1000 kcal)	0.6	(0.6–0.7)	0.6	(0.5–0.7)	0.6	(0.6–0.7)	0.7	(0.6–0.8)	0.469
Zinc	(mg/1000 kcal)	4.4	(4.1–4.8)	4.4	(4.0–4.8)	4.4	(4.2–4.9)	4.5	(4.2–5.0)	0.821
Vitamin C	(mg/1000 kcal)	51.3	(42.6–67.9)	51.7	(42.9–62.7)	50.6	(41.7–66.8)	64.0	(40.8–76.5)	0.912
Calcium	(mg/1000 kcal)	311	(256–370)	309	(244–367)	323	(258–374)	297	(243–330)	0.495
Dietary fiber	(g/1000 kcal)	6.6	(5.7–7.8)	6.6	(5.8–7.8)	6.7	(5.7–7.8)	6.6	(5.1–8.3)	0.938
Protain	(% energy)	14.6	(12.8–16.2)	14.4	(12.8–16.5)	14.7	(12.8–16.0)	16.0	(14.3–17.1)	0.120
Fat	(% energy)	28.9	(25.3–33.3)	28.5	(23.9–33.9)	28.2	(25.3–32.4)	32.0	(30.5–35.4)	0.042
Carbohydrate	(% energy)	54.6	(50.0–59.7)	55.6	(49.9–61.9)	55.7	(51.2–59.6)	50.3	(49.5–52.1)	0.026

IQR, Interquartile range. Kruskal–Wallis test.

## Data Availability

These will be made available upon reasonable request from the corresponding author M.H.
